# Success with dorsal root entry zone lesioning after a failed trial of spinal cord stimulation in a patient with pain due to brachial plexus avulsion

**DOI:** 10.1097/PR9.0000000000000973

**Published:** 2021-11-22

**Authors:** Lucia Lopez, Andrei D. Sdrulla

**Affiliations:** Department of Anesthesiology and Perioperative Medicine, Oregon Health and Science University, Portland, OR, USA

**Keywords:** Brachial plexopathy, Neuropathic pain, Spinal cord stimulation, Neuromodulation

## Abstract

Pain caused by brachial plexopathy (BP) represents a challenging clinical problem with few effective therapeutic options. Here, we present a patient with severe, painful BP after a high-impact motor vehicle accident who failed conservative treatments. A trial of cervical spinal cord stimulation was completed using multiple waveforms (tonic, BurstDR, and 10 kHz) over 14 days with only 30% to 40% pain reduction. Subsequently, he underwent dorsal root entry zone lesioning with a significant decrease in his pain 1 year later. Surgical exploration revealed extensive damage and avulsion of his cervical roots that was not observed on a previous brachial plexus magnetic resonance imaging. We discuss the etiology and diagnosis of traumatic BP, possible reasons for the failed spinal cord stimulation trial, and implications for management.

## Abstract 2

We report a case of successful dorsal root entry zone lesioning after a failed trial of cervical spinal cord stimulation in a patient with severe pain due to brachial plexus avulsion.

## 1. Introduction

Brachial plexopathy (BP) develops most commonly because of high-speed collisions involving motor vehicles.^31^ Many patients with BP develop severe, intractable neuropathic pain that is resistant to therapies and is associated with substantial morbidity.^6,21^ In general, the severity of pain correlates with the extent of the nerve trauma.^6,7,14^

Although nerve injuries were historically classified depending on the extent of damage to nerve structures, specific classification schemes have been proposed for the brachial plexus based on the mechanisms of injury and anatomical locations of the lesions.^16,20,26^ An important distinction concerns the relation of the lesion to the dorsal root ganglion because preganglionic lesions are associated with more severe pain that is refractory to treatments.^21^ Surgical exploration of the brachial plexus remains the gold standard for establishing the location of the injury; however, this is not feasible in most patients. Imaging techniques such as magnetic resonance imaging (MRI) and computed tomography have been used extensively to characterize the location and extent of injury, although with limited accuracy.^15^

Brachial plexopathy treatments initially focus on reversing the underlying cause or surgically repairing the injured nerves. Chronic pain management is often challenging, and multiple approaches have been used, including medications, neuromodulation, and ablative surgical interventions.^27^ Neuromodulation approaches, such as spinal cord stimulation (SCS) and peripheral nerve stimulation, are regarded as appealing as they are minimally invasive, durable, reversible, and associated with low rates of severe complications.^10,11^ Early case series documented low success rates with SCS.^28,30^ However, recent publications reported substantial pain relief with cervical SCS, possibly due to improved stimulation devices and novel waveforms.^13^ In patients with severe pain and weakness, dorsal root entry zone (DREZ) lesioning is recommended, although many patients are hesitant to proceed because of the associated morbidity intrinsic to the ablative nature of the procedure.^1,29^

Here, we present a patient with preganglionic BP caused by trauma who failed medications and a trial of cervical SCS but responded to DREZ lesioning.

## 2. Case report

A 71-year-old Hispanic man presented with intractable severe right arm pain after an all-terrain vehicle accident 3 months earlier. The patient developed right upper extremity weakness, pain, and loss of sensation immediately after the accident. He described severe constant burning pain and episodic electric shock–like pain with a numerical rating scale of 7-9 of 10.

On examination, sensation was absent on the right forearm, hand, and fingers. He could generate trace movements in his fingers and none in the rest of the arm and shoulder.

He suffered multiple injuries at the time of the accident, including rib and scapular fractures and C6/7 right transverse process fractures, treated nonoperatively. A cervical MRI obtained at the time of the accident described a pseudomeningocele within the right C7-T1 neural foramen with nonvisualization of the right C8 nerve root concerning for avulsion. A brachial plexus MRI (3T; sequences T1, T2, STIR, and SPAIR) performed 6 months later revealed hyperintensity of the entire right brachial plexus without evidence of root avulsion; the pseudomeningocele was no longer present. The MRI report mentioned that the roots of the brachial plexus were well visualized bilaterally. A nerve conduction study was consistent with severe pan-BP.

**Figure 1.**
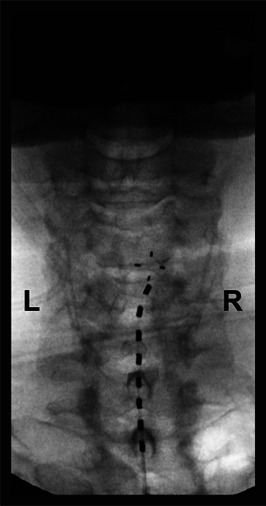
Anteroposterior views of the final spinal cord stimulator lead position at the time of placement.

**Figure 2.**
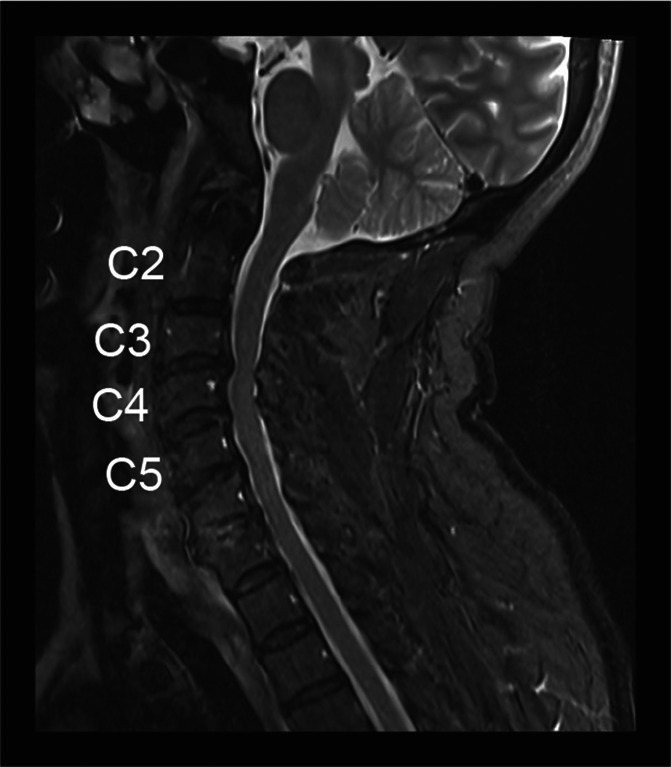
Sagittal cervical spine STIR image showing canal stenosis at C3-4 and C4-5.

Multiple medications were tried without sustained benefit, including short-acting and long-acting opioids, pregabalin, baclofen, amitriptyline, and duloxetine. Physical therapy did not improve his pain. The patient underwent a trial of cervical SCS 8 months after the accident. An 8-contact percutaneous lead was placed slightly to the right of the midline at the top of the C5 vertebral body (Fig. 1); paresthesia mapping revealed coverage of the entire right shoulder, arm, and hand. Only one lead was placed because of the observed degenerative changes in his cervical spine on MRI (Fig. 2), including mild to moderate canal stenosis at C3-4 and C4-5. An external stimulator was used to deliver BurstDR stimulation,^9^ and programming was adjusted based on paresthesia testing 3 days after lead placement. He reported minimal pain relief at the end of the 5-day trial. Tonic stimulation was then attempted for 2 days, but paresthesias were not tolerated because of discomfort. Seven days after lead placement, the external stimulator was switched to a device capable of delivering 10 kHz stimulation, and paresthesia mapping was repeated to confirm lead positioning. The patient reported 30% to 40% pain reduction at the end of a 7-day trial with 10 kHz stimulation; implantation was not offered.

He underwent DREZ lesioning (C3-T1) about 2 years after the initial injury. He reported 75% pain reduction immediately, and 1 year after the DREZ procedure, with substantially improved pain scores (average numerical rating scale 4/10). During surgery, numerous dorsal and ventral root defects were documented from C5-T1, including the absence of the C6 and C8 dorsal and ventral roots.

## 3. Discussion

Here, we present a patient with traumatic BP who failed conservative treatments, including neuropathic medications and physical therapy. The patient had a negative SCS trial despite appropriate paresthesia mapping, extensive programming efforts, and the use of modern stimulation waveforms. Dorsal root entry zone lesioning 2 years after the initial accident provided substantial pain relief.

It is critical to distinguish between preganglionic and postganglionic lesions in patients with BP because preganglionic pathologies correlate with a worse prognosis. Furthermore, surgical interventions (nerve grafting vs transfer) depend on the location of the lesion.^21,30^ Our case is unique in that the initial cervical MRI visualized a pseudomeningocele and possible root avulsion at C8; although a follow-up study 6 months later found no evidence of root pathologies, subsequent intraoperative exploration during the DREZ procedure—the gold standard for assessing root injuries^34^—described complete avulsion at C6 and C8, with abnormalities throughout the entire plexus. Therefore, the avulsed C8 root corresponded to the pseudomeningocele described in the initial MRI. A recent study examined the accuracy of MRI for predicting traumatic root avulsions and reported a negative predictive value of 81%.^32^ Thus, of 5 cases reported not to have avulsion, 1 will have it—as in our case. The predictive value of pseudomeningocele for avulsion was 68%.^32^ Recent studies using novel sequences and stronger magnets demonstrated high accuracies (>90%) for detecting root avulsions.^23,34^ Our patient had a brachial plexus MRI using a 3T magnet and standard sequences, suggesting that additional research is needed to define the optimal techniques for detecting preganglionic injuries in practice.

Our patient obtained 30% to 40% pain relief during the SCS trial and was not offered implantation. The lack of efficacy observed with SCS could have been due to technical factors such as suboptimal lead position (despite paresthesia testing) and/or stimulation parameters. Multiple reports have described substantial pain relief in patients with preganglionic BP using cervical SCS.^13^ One study found initial success with conventional SCS followed by loss of efficacy; however, pain relief was rescued by switching to high-frequency (10 kHz) SCS at C2/3.^17^ We placed a single electrode at C5 because of the patient's unfavorable anatomy (Fig. 2). Although electrode placement and paresthesia mapping are critical for tonic stimulation, it is less clear for BurstDR and 10 kHz waveforms and less so in the cervical spine.^3,8,12,22^ Our outcome suggests that high cervical placement should be considered for BP, if feasible. Future studies are needed to characterize optimal lead placement for different pathologies and stimulation waveforms.

It has been suggested that the pain relief threshold required for implantation in patients with BP should be lowered to less than 50%.^13^ This change would account for the severe, refractory pain usually associated with BP and would be more aligned with clinically significant changes in pain scores for this population. Our patient would have been offered implantation with this adjustment. However, in our opinion, this should be addressed further because SCS tends to lose efficacy over time, and there is a strong placebo effect early on.^2,4^

The extensive deafferentation injuries observed during the DREZ surgery likely contributed to the refractory nature of our patient's pain.^21^ Spinal inhibitory circuits reside in the superficial layers of the dorsal horn, and root avulsion results in both damage to these circuits and inability of SCS to engage them.^19,25,30^ Our patient obtained significant relief after DREZ lesioning. This procedure entails surgical destruction of both Lissauer tract and superficial dorsal horn structures, disrupting transmission and processing of nociceptive and other inputs.^5^ Therefore, DREZ lesioning attenuates pain by diminishing afferent drive and preventing pathological dorsal horn processes that amplify pain and produce sensitization.^33^ Multiple reports described substantial benefit after DREZ surgery in patients with avulsion.^18^ Our patient reported 75% reduction in pain 1 year after surgery, consistent with previous reports, suggesting that DREZ should be part of the treatment algorithm for severe BP.^1,24,29^

In summary, we present a case of traumatic BP who failed conservative treatments and a trial of cervical SCS but obtained significant benefit from DREZ lesioning. The initial imaging did not reveal his extensive preganglionic pathology, which likely contributed to the refractory nature of his pain. Our report hopes to raise awareness of the need for further research into the diagnosis and treatment of preganglionic BP–related pain.

## Disclosures

The authors have no conflicts of interest to declare.
